# Contributions of medicinal plants to the Gross National Happiness and Biodiscovery in Bhutan

**DOI:** 10.1186/s13002-015-0035-1

**Published:** 2015-06-03

**Authors:** Phurpa Wangchuk, Tashi Tobgay

**Affiliations:** Manjong Sorig Pharmaceuticals, Ministry of Health, Thimphu, Bhutan; Centre for Biodiscovery and Molecular Development of Therapeutics, Queensland Tropical Health Alliance, James Cook University, Cairns Campus, QLD 4870 Australia; Khesar Gyalpo University of Medical Sciences of Bhutan, Thimphu, Bhutan

**Keywords:** Medicinal plants, Bhutanese traditional medicine, Health care services, Gross national happiness, Biodiscovery, Environmental conservation, Socio-economic development

## Abstract

**Background:**

The medicinal plants and the associated Bhutanese traditional medicine (BTM) are protected by the country’s constitution and receive both government support and acceptance by the wider public. More than 1000 medicinal plants are described in the BTM but currently collects only 300 species for daily formulations of BTM. These medicinal plants have been one of the drivers of the ‘Gross National Happiness (GNH)’ and biodiscovery projects in Bhutan. However, no review covering the systematic evaluations of the contributions of medicinal plants and the BTM to the GNH and biodiscovery exist till date.

**Methods:**

This paper, therefore addresses this information gap. It is based on the review of the existing traditional and scientific literature, government websites and policy documents. The descriptions and discussions of the paper is straightened, authenticated and enhanced by the data collected through the informal discussions with the BTM practitioners and also through the authors’ many years of practical observations of the impact of the medicinal plants programs and the BTM practices in Bhutan.

**Results:**

This paper found the following: a) the medicinal plants generates income to the farmers elevating their living standard and the economic status, b) it serves as the bulk ingredients of the BTM facilitating the provision of free traditional health care services to the patients, c) helps the conservation of medicinal plants and their pristine environment through recognition of their spiritual, social and economic values, d) preserves the rich BTM cultural heritage, and e) guides the biodiscovery projects based on their ethnobotanical information. The paper also identified the challenges and research gaps, and recommends appropriate strategies that can help secure the sustainable future of the medicinal plants, the BTM and the biodiscovery projects.

**Conclusions:**

The medicinal plants play significant role in the country’s biodiscovery projects and the internationally renowned development policy of ‘Gross National Happiness’.

## Background

The biological resources such as plants, animals and microorganisms exhibit bewildering properties wherein their whole parts or derivatives can be used for social, economic, environmental, traditional medicines, scientific and chemotherapeutic purposes. Plants are used as bulk ingredients in traditional medicines (TM) which provides the primary health services to 80 % of the world’s population [1]. Besides playing indispensable role in the primary health care, the medicinal plants has been also one of the important source of modern drug discoveries. This is mainly because their long history of clinical uses enhances the hit rate of a new drug lead candidate. An analysis by Fabricant and Farnsworth [2] on the origin of the drugs developed between 1981 and 2001 showed that 80 % of 122 plant-derived drugs were related to their original ethnopharmacological uses. Between 2000 and 2005, about five medicinal plant-based drugs were introduced in the United States market and another seven plant-derived compounds are currently in clinical trials around the world [3, 4].

It is estimated that about 50,000 plant species are used in the TM worldwide with majority of them being in Asian medicines [5, 6]. These medicinal plants contain valuable ethnobotanical information that could navigate new drug discoveries. Table 1 shows a compiled list of the availability of higher plants and the usage of medicinal plants by the selected countries [5–9]. It is indicated that Indians utilize maximum 20.0 % of their plant flora as medicinal plants which is followed by China (18.9 %), Vietnam (17.1 %), Sri Lanka (16.5 %) and Thailand (15.5 %). While nations like the USA, Australia, Indonesia and Malaysia have a high number of plant species, their utilization as medicinal plants are low. Australia have utilized 7.8 % of its higher plant flora as medicinal plants and this knowledge remains in Aboriginal communities [7].

**Table 1:** Number of plant flora and the medicinal plants reported from selected countries [5–9]

Country	Higher plant species	Medicinal plant species	% of medicinal plants
Africa^a^	45,000	5000	11.1
Australia	19,324	1,511	7.8
China	26,092	4,941	18.9
Bhutan	5,603	600	10.7
India	15,000	3,000	20.0
Indonesia	22,500	1,000	4.4
Malaysia	15,500	1,200	7.7
Nepal	6,973	700	10.0
Pakistan	4,950	300	6.18
Philippines	8,931	850	9.5
Sri Lanka	3,314	550	16.5
Thailand	11,625	1,800	15.5
USA	21,641	2,564	11.8
Vietnam	10,500	1,800	17.1

^a^All African countries combined

**Fig. 1:**
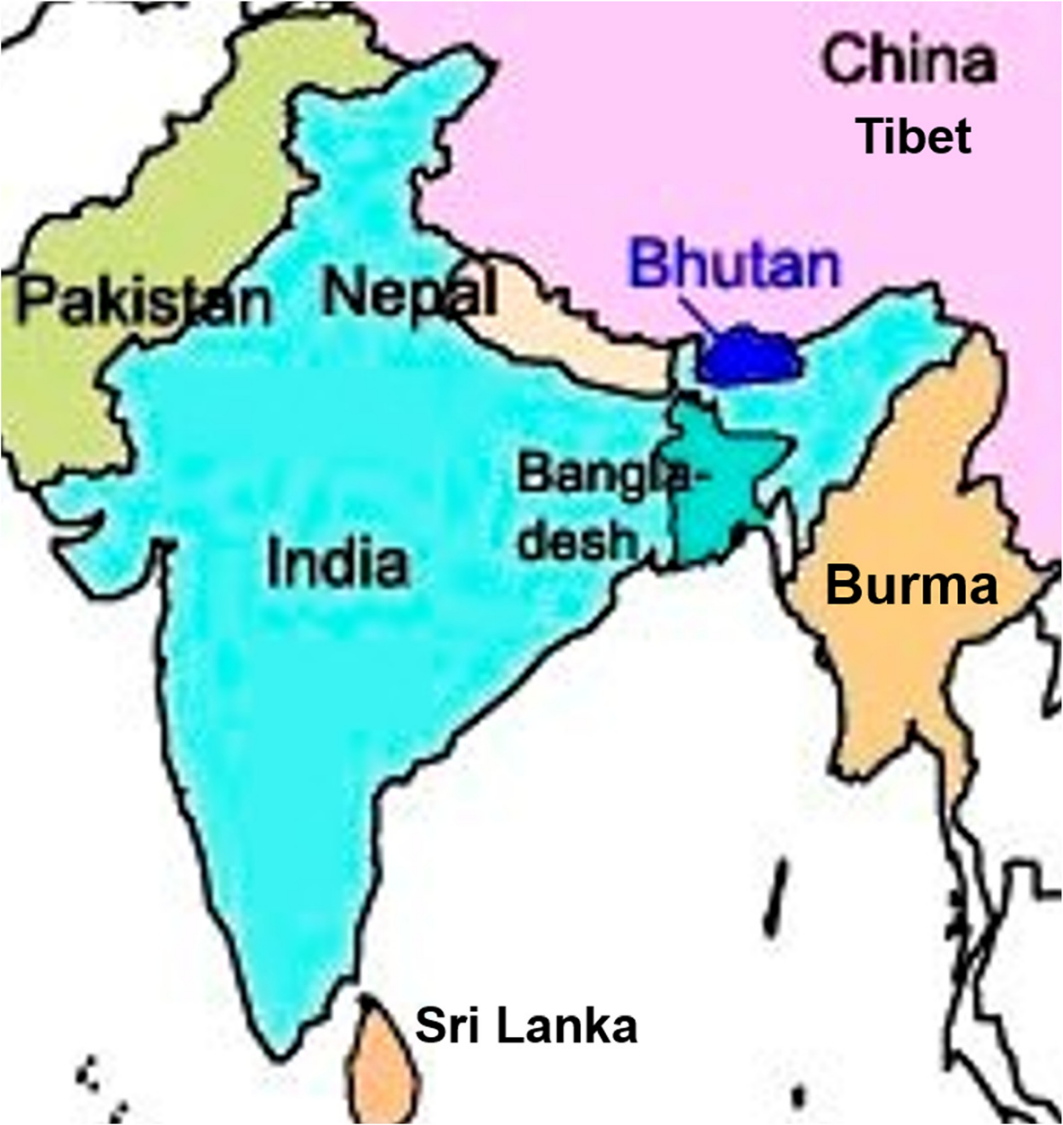
Map of Bhutan [34]

In Bhutan, more than 5603 higher plant species are recorded and most of them are believed to have medicinal properties [10]. In ancient times, due to abundance of medicinal plants, Bhutan was known by many synonyms as ‘*bruk-tsan-dhen-b.kod-pai-rgyal-khab*’ (translated as the dragon kingdom of bountiful sandalwood) and *lho-jong sman-jong* (translated as the southern land of medicinal plants) [11]. Bhutan, which is nestled in the eastern Himalayas between India in the south and Tibet (China) in the north (see Fig. 1. Map of Bhutan), is a small mountainous country with the total area of 38,394 sq. km and a population of 658,888, [12]. The notable differences in altitude ranging from 150 m above sea level (masl) in the sub-tropical south to 7500 masl in the alpine north [10], has facilitated the development of diverse ecological zones with rich biodiversity. With 72 % of the country still under forest cover, it is considered as one of the ten global biodiversity-hotspots *par excellence* in the world. The economy of a country is mainly based on hydroelectricity, tourism, mining and renewable natural resources (RNR) including agriculture, livestock, forestry and medicinal plants.

The impenetrable forest and ravines and the interlocked high mountains have secluded the inhabitant of different valleys, which facilitated the development of unique culture and traditions including traditional medical cultures. There are two types of traditional medical systems: an orally transmitted folklore medicines and the scholarly *g.so-ba-rig-pa* medicine. While the folklore medicines (also called the local healing practices) play important role in the spiritual health of the communities, their use of medicinal plants are considered negligible. On the other hand, the *g.so-ba-rig-pa* medicine which is popularly known as the Bhutanese traditional medicine (BTM) has been integrated with the mainstream health care system of the country. Although, this medical system has been adapted from the larger corpus of scholarly Tibetan medicine, differences can be observed in the golden needle therapy methods and the utilization of medicinal plants in the formulations [13]. The BTM and the Tibetan textbooks describes more than 1000 medicinal plants and 600 of them that grows in Bhutan have been already identified [5]. Out of this, 300 of them are currently used on a daily basis at the Manjong Sorig Pharmaceuticals under the Ministry of Health in Bhutan for preparing multi-ingredient formulations [1].

Today, the preservation of biodiversity hotspot and rich culture and traditions including BTM forms basis of the country’s novel development philosophy called ‘Gross National Happiness’ (GNH). The GNH was espoused by the fourth King of Bhutan, Jigme Singye Wangchuck, in 1972 as a higher level development philosophy than the conventional development principles of Gross Domestic Products (GDP) [12]. This GNH philosophy is based on the belief that all human beings aspire happiness and that the true development of human society is the measure of the collective happiness of people achieved through balancing spiritual wellbeing with economic progress. The BTM and medicinal plant programs play important role in improving the wellbeing and the socio-economic status of the people in Bhutan. The medicinal plants have empowered farmers through income generation by collecting the selected plants on an annual basis. Indeed, the medicinal plant program is one of the important sustainable development initiatives of Bhutan. It has been generating happiness for farmers, yak herders, brokers, herbal industries and many stakeholders. Therefore, this review paper describes the role of the BTM and medicinal plant programs in achieving GNH and in navigating the biodiscovery projects in Bhutan. The paper also identifies research gaps, and suggest perspectives for future research.

## Methodology

The information on the medicinal plants and the BTM were extracted from published literature and from discussions with practitioners of BTM. The published literature included scientific journals, books, reports from national, regional and international organizations, thesis, conference papers, policy documents, constitution, and the related government and non-government websites. Literature was searched on Google Scholar, SciFinder Scholar and the PubMed Central using specific search terms such as “Medicinal Plants”, “Bhutanese Traditional Medicine”, “Cordyceps”, “Gross National Happiness”, “Biodiversity”, “Biodiscovery”, “Bioprospecting”, “Integration”, Primary Health Care” and “Bhutan”. The data were assessed and compiled. The results and discussions were enhanced using many years of our own personal experience with the BTM and the medicinal plants collection programs. A series of plant surveys to numerous places and visits to two main medicinal plants collection centres, Lingzhi (North Bhutan) and Langthel (Central Bhutan) were made during those years. The first hand field experiences provided considerable enrichment of information collected from the literature.

## Results and discussions

### Conceptual framework of GNH and its relationship with the BTM and medicinal plants

**Fig. 2:**
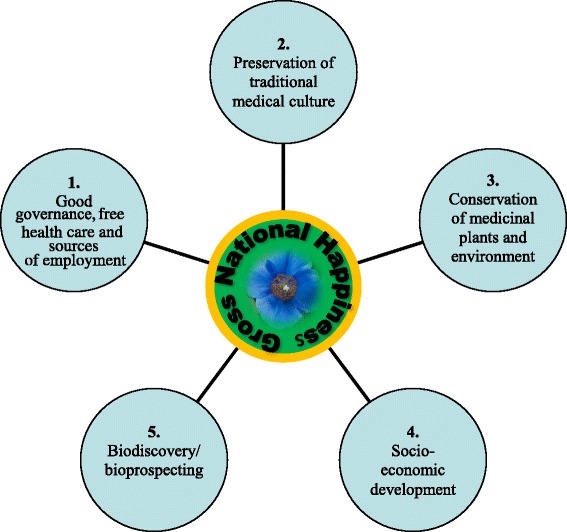
Five pillars of medicinal plants that are contributing to GNH in Bhutan

The GNH-based development philosophy of Bhutan has inspired many multinational politicians, philosophers, scholars, economists and the United Nations (UN) due to its holistic concept and approach to development. Professor Jeffrey D. Sachs [14], who is one of the world’s most influential living economists and a leader in sustainable development at the Earth Institute at Columbia University, believes that this GNH philosophy can help recreate a sensible economic life and happiness in America and the rest of the world which are afflicted by rapid urbanization, mass commercial media, global capitalism, relentless pursuit of GDP, environmental degradation, and failing health care system. The GNH concept is based on the realization that collective happiness of the society is more important than material growth alone, which has been explained and measured by its four pillars and nine domains [12]. The four pillars includes: good governance, sustainable socio-economic development, cultural preservation, and environmental conservation. The pillars are further branched into nine domains: psychological wellbeing, health, education, time use, cultural diversity and resilience, good governance, community vitality, ecological diversity and resilience, and the living standards [15]. While the BTM or *g.so-ba-rig-pa* medical tradition falls under the pillar of preservation of culture and traditions, its related medicinal plant programs encapsulates two GNH pillars: sustainable socio-economic development and environmental conservation (Fig. 2).

The preservation and development of the BTM and its integration with modern medicine has broadened the health care choices and benefited the people. While it has huge potential to create more jobs and businesses, the future of BTM culture and services rely on knowing to strike a fine balance between the demand for BTM services and the sustainable supply of medicinal plants. If sustainable supply of medicinal plants is to be achieved, matching the policies, good governance and the farmers’ participation to the BTM and medicinal plant programs are essential. The existing policies and good governance practices that favored the growth and development of BTM is discussed in details in the section below.

### Good governance, BTM promotion and the creation of employment

The good governance system established by the successive kings, governments, and the BTM practitioners have played significant role in the preservation and development of this rich ethnomedical tradition and the sustainable utilization of the medicinal plants in the country. The BTM and the medicinal plants are safeguarded by the constitution and government policies. Article 9 of the constitution of the Kingdom of Bhutan states that “The state shall provide free access to basic public health services in both modern and traditional medicines” [16]. Bhutan 2020: A Vision for Peace, Prosperity and Happiness [17] also states that, “We must continue to provide a place for traditional medicine in our health care system as it embodies knowledge, draws upon the nation’s rich biodiversity, and provides an alternative health care choices for those who seek one”. The national health policy of the Ministry of Health [18] states that, “Focused efforts shall be directed towards making the BTM, the centre of excellence in providing quality traditional medical services including wellness centre that is recognizable at an international level and that the identification, demarcation and protection of areas rich with medicinal plants be managed judiciously by the relevant district administration in conformity with the conservation regulations of the Ministry of Agriculture and Forestry”.

The past governance and the developments related to the BTM and medicinal plants in Bhutan has been described previously [19–21]. These historical documents highlights three important spiritual figures that have prominently influenced and contributed to the beliefs, faith, philosophy, inception and development of the traditional medical system in the country. They are: Sangay sman-lha (Medicine Buddha, 2500 B.C), Guru Padma Sambhava (Tantric Buddhist saint of the 8th Century A.D), and Zhabdrug Ngawang Namgyal (Bhutanese political and religious leader of 17th Century A.D). The epic transformations of BTM was ushered by the third king, Jigme Dorji Wangchuck, who integrated the BTM with biomedicine in 1967. The fourth king, Jigme Singye Wangchuck, propounded the GNH philosophy in 1972 which safeguarded the BTM and ensured its continuity. The BTM evolved from a single traditional medical dispensary in 1967 to one of the complex and bigger organization called the Institute of Traditional Medicine Services (ITMS) in 1998. Today, there are three separate sections responsible for the preservation, promotion and development of BTM. The Faculty of Traditional Medicine (FTM) under the Khesar Gyalpo University of Medical Sciences of Bhutan (KGUMSB) offers the *Drungtsho* and *sman-pa* courses [22]. While the term *Drungtsho* is locally used for Traditional physicians after completing 5 years of degree in BTM, the term *sman-pa* is used for traditional clinical assistant after obtaining three years of diploma courses in the BTM. The Manjong Sorig Pharmaceuticals (MSP) conducts research and manufactures more than 100 medicines for distributing to Traditional Medicine Hospitals (TMH) in the country. The TMH provides free primary health care services to the people through 48 traditional medicine units that are established wide across the country. Analyzing the current organizational and human resource structure revealed that these three organizations (FTM, MSP and TMH) together provide employment to a total of 212 trained people [23, 24] to cater for the traditional health care services. In addition, many farmers, yak-herders and business people are involved in the collection, trade and distribution of medicinal plants. The FTM has 61 enrolled students as of 2014 [22]. As the BTM services and related herbal industry expands, it is expected to create more jobs in future.

### Preservation of BTM through incorporation of scientific protocols

The ITMS was given the initial mandates to preserve, promote and develop this rich traditional medical heritage. The practitioners believed that to successfully preserve this age old tradition, the accessibility to the BTM medicines and the people’s acceptance were crucial. The people’s respect towards BTM was eminent as this medical tradition was supposed to have originated from Gautama Buddha’s teaching. However, as the communities became more educated, people’s respect and acceptance relied on scientific evidences of the BTM. Between 1980s and 1990s, numerous funding bodies including World Health Organization, Italian DISVI project and two successive European Union projects (Phase I in 1994–1998; Phase II in 2006–2009) were brought in to help improve the quality of BTM and the sustainability of medicinal plants as well as achieve long term preservation goal. Under these projects, unprecedented progress were made in terms of the development of the infrastructures, research and quality control protocols, manufacturing process and service delivery system.

While modern scientific approaches including the Good Manufacturing Practices (GMP), Good Research Laboratory Practices (GLP), Good Collection Practices (GCP), Good Dispensing Practices (GDP), Standard Service Delivery and Treatment Practices (SSDTP), the Total Quality Control System (TQCS), and the Adverse Drug Reaction Surveillance and Reporting System (ADRSRS) were introduced, it was made sure that the original BTM practices were retained, preserved or slightly adapted to current needs. For example, in the quality control parameters of the raw materials, the ancient/traditional methods to identify, evaluate and grade the quality of medicinal ingredients through the macroscopic, organoleptic property and characteristic observation were retained and preserved. To supplement these ancient quality parameters, microscopy, physiochemical (total ash value, moisture content, loss on drying, essential oil content) and comparative thin layer chromatography analysis were introduced [12]. Similarly, in case of the finished products, their formulations and dosages were adapted and adjusted from the traditional dose measurement by: a) a small spoon if it is powder, and b) traditionally prepared pills. To make it more palatable to the patients, modern capsule and tablet dosage forms were introduced. While the ancient quality parameters including observation of the appropriate color, aroma, taste, hardness and the solubility of the products were preserved; the modern test protocols such as the measurement of pH values, friability, disintegration and bulk density, and packaging with appropriate materials were introduced.

The laboratory quality parameters are important but the practitioners and the MSP believed that the quality, safety, and effectiveness of the BTM depended largely on the quality of their raw materials and how they are handled through collection and production processes. Therefore, much emphasis has been given to the ancient practices of the *g.ches-pa’i-yan-lag-b.dun* or the seven branches of quality practices which has been dealt elsewhere [12]. In terms of traditional medical service delivery, everything is practiced as prescribed in the traditional medical texts including the BTM’s codes of disease classification and health problems, the standardized traditional treatment and therapy guidelines, and the counseling of patients. The modern GDP and ADRSRS were recently introduced to supplement and improve the quality of the services. The need was also felt to improve the traditional teaching and learning methods. Recently, the FTM have incorporated the modern curriculum while preserving most of its ancient methods including memorization and examination process.

Thus, preservation of this traditional medical heritage can be claimed as a success. A recent study by Lhamo and Nebel [25] on the perception and attitudes of the Bhutanese towards the BTM health care services reported that 51 % of 155 respondents of the study (only one study area) were being treated by the BTM practitioners and about 83 % of them were satisfied with its quality and the health services. This finding suggested that the BTM will continue to have its role as one of the primary health care provider in the years to come.

### Conservation of medicinal plants and their pristine environment

Our review found no separate and specific act and policy for the conservation of medicinal plants. However, the Biodiversity Action Plan (BAP)-2009 [11] and the Health Policy-2010 [18], provides explicit description of the importance of the medicinal plants and their need for protection. The environment in general is regarded as sacred by the people because they believe local deities reside in the pristine mountains, cliffs, riverbanks and meadows. Even more so, the respect for the medicinal plants and their environment are overwhelmingly higher. The farmers regard it as a boon to have a patch of land where medicinal plants grow abundantly. It is considered a sin to defecate and pollute these areas and is believed to earn good merits through collection of the medicinal plants for the sick people. Thus, these beliefs have instilled the people with the natural and ethical conservation mind-set. In addition, the income they can generate from the cultivation and collection of medicinal plants has strengthened their respect towards it.

The medicinal plants in Bhutan are categorized into two types depending upon where they grow. Those medicinal plants which grow in the alpine environment are known as the High Altitude Medicinal Plants (HAMP) and the other growing in the temperate and tropical environment are called the Low Altitude Medicinal Plants (LAMP). With the assistance from the European Union, the medicinal and aromatic plants projects were implemented by the Ministry of Agriculture by establishing a Medicinal and Aromatic Plants Section (MAPS). This MAPS carries out the wild medicinal plant surveys, cultivation trials, and identifies the markets other than the MSP. The MAPS also in collaboration with the MSP conducts farmers’ training on the sustainable collection of the wild species of medicinal plants. To conserve the medicinal plants that are not common, community herb gardens were established in Lingzhi for the high altitude plants and Lingmithang in Eastern Bhutan for the low altitude plants. Domestication and cultivation trials are constantly performed at Yusipang in Thimphu.

Most of the HAMP grow within the protected biological corridors and the national parks which are under the vigilance of forest rangers. The MSP has to seek a blanket approval for the required amount of the medicinal plants to be collected on a yearly basis from the Department of Forest and the concerned National Parks. The BTM practitioners believe that the current utilization of the medicinal plants are under sustainable range. However, the farmers or the collectors expressed that the growth of the medicinal plants have been shrinking in and around the collection centres. This was due to impacts of climate change and also as a result of the persistent collection practices for more than 45 years. Realizing this issue, the MSP have identified the alternative collection centres at Tshampa in Northern Bhutan and Dagala in Western Bhutan for HAMP, and Goshing in Southern Bhutan for the LAMP. The risk factors for the sustainable use of these medicinal plants in Bhutan has been discussed elsewhere [26].

### Socio-economic development through collection and sale of medicinal plant products

**Fig. 3:**
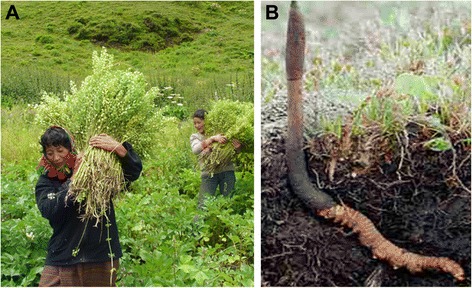
**a**. Farmers collecting medicinal plants (courtesy: P.W collection), **b**. *Cordyceps sinensis* (Courtesy: pixgood.com)

Medicinal plants are the bulk ingredients used in the multi-ingredient BTM formulations. As a result, the medicinal plants collection program has become one of the main income generating activities for farmers in Bhutan (Fig. 3a). Each household are given yearly training for the sustainable collection of medicinal plants in their localities. They are distributed with the plant list to be collected and are paid mostly on a per kilogram wet-weight basis. The price of the plants are fixed and revised by the government after every two years or as and when deemed necessary in consultation with the farmers. The annual collections of about 116 HAMP species are carried out in between May-August and about 92 LAMP species in between December-February. Between 2006 and 2008, the ITMS procured about 13 metric tons of dried medicinal plants from the farmers, which in monetary terms amounted to Nu. 4 million (USD 97,600 approximately), and the return from the sale of these materials and their finished herbal products amounted to Nu. 8 millions [27]. The finished herbal products manufactured and marketed by the MSP comprises 100 Essential Traditional Prescription Medicines (ETPM) and 18 commercial products including different types of health promoting herbal teas and cordyplus products, varieties of incense and spiritual merchandises, massage oil and mind soother balm.

The recent 2014 data was not reported in the literature but the reliable sources and the inventory list of medicinal plants maintained by the MSP indicated that this collection trend remained almost similar over the last 5 years. While all the HAMP were collected by the yak-herders from within Bhutan, more than 64 LAMP were collected by the farmers and the remaining 28 species were supplied by the herbal suppliers who generally import them from India. Our earlier study on LAMP [13] found that as many as 16 of these imported species grow abundantly in Bhutan and has potential to generate good income for farmers through their cultivation and collection program. This medicinal plant activities have had cascading effects on many stakeholders including traditional medicine hospitals (TMH), NITM, MSP, MAPS, farmers, semi-nomadic yak herders, herbal markets, traders, and the patients. If hydropower projects drives the country’s macroeconomics, the medicinal plants programs can be considered the microeconomics that improves farmers’ socio-economic status. Farmers have expressed that due to the medicinal plant collection program, they could afford good houses, horses, household amenities and could send their children to schools. Beside medicinal plant collection, these same farmers also earn income by working as a porter or engaging their horses for transporting the medicinal plants and the eco-tourists. Thus, medicinal plant programs have brought in the wealth, prosperity and the happiness to the farmers. It has also improved the community dynamics, vitality and renewed their sense of belongingness to the villages and medicinal plants collection centres thereby preventing rural–urban migration.

One of the high value insect-fungi (Fig. 3b), which is considered medicinal plant in Bhutan, is *Cordyceps sinensis*. Marketing of *Cordyceps* was legalized in 2004 and has been a financial boon for many highlanders from the districts of Bumthang, Gasa, Wangdue Phodrang, Trashi Yangtse, Lhuentse, Paro, and Thimphu. For sustainability issues, every household is entitled to three permits each costing Nu. 350 that has the validity for a month. The assessment on the impact of this medicinal plants on the livelihood of the collectors from the selected five blocks or sub-districts of Bumthang and Wandue Phodrang was carried out by the Ugyen Wangchuck Institute for Conservation and Environment (UWICE) [28]. This UWICE assessment reported that in 2012, about 394 *Cordyceps* collectors from the two collection areas were involved and that they earned about Nu. 57 million (about $ 12 million) from 2004 to 2009 which translated to each household earning about Nu. 0.14 million since they started collecting. Majority of this income was used for purchasing food, household items, clothing, livestock, land and vehicle; constructing new houses or to repair old ones; and for their education [28]. In 2013, the Highlanders together auctioned about 685 kgs of *Cordyceps*, the highest quantity so far. Wangdue farmers collected the largest amount of about 373 kgs, which fetched them more than Nu. 1.14 million a kg and the Gasa farmers collected 101 kgs to earn Nu. 1.21 million a kg, the highest price that year [29]. The overall *Cordyceps* yield decreased slightly in 2014, with 555 kgs of *Cordyceps* collected and sold in the auction yard for Nu 470 million [30]. Gasa highlanders got the best price with an auction price of Nu 1.326 million/kg (about $26,520) and the government earned a total of Nu. 5.64 million as royalties from these sales [30]. These records are based on the sale proceeds of those collectors who took part in the *Cordyceps* auction yards and does not account for those who did not take part in the auction or who sold their collections directly to buyers.

As the herbal industries including Bio-Bhutan, incense factories and other non-wood related small scale industries are beginning to grow, the demand for other medicinal plants is also expected to rise. Number of incense factories have doubled over the years thereby increasing demand for the medicinal plants such as *Nardostachys jatamansi* (*Pang-poe*), *Aquillaria malaccensis* (*Agaru*), *Carthamus tinctorus (Gur-gum)*, *Juniperus* species (*Shug-pa*), *Canarium strictum* (*spoe-d.kar*), *Saussurea lappa* (*Ru-rta*), *Inula racemosa* (*Ma-nu*), *Pterocarpus santalinus* (*Tsan-den d.mar-po*) and *Rhodendron* species (*Balu* and *Sulu*). In addition, many medicinal plants have huge potential for the international market including the pharmaceuticals and cosmetic industries. However, Bhutan must be careful about mass commercialization of medicinal plants as it can have irreversible negative effects on the resilience and vitality of the plant species, and its environment. Lessons can be learnt from Nepal, India and Tibet (China) where the wild medicinal plants are being over-farmed.

### Biodiscovery potential of medicinal plants and current status

The strong tradition of herbal remedies within the BTM forms a unique opportunity for biodiscovery. The Bhutanese flora is characterized by an outstanding bio-diversity and a large number of endemic species, many of which forms part of the BTM pharmacopoeia. It would be worthwhile to explore and extract that rich ethno-botanical information so as to aid and guide the modern drug discovery programs in the right direction. Most of the Bhutanese plants are not studied before, and since some of them grow in extreme climatic conditions, they may be hosting cures for diseases including HIV/AIDS, Cancer and other infectious diseases.

Realizing the huge potential of the medicinal plants, the MSP and the National Biodiversity Centre (NBC) have initiated sporadic biodiscovery projects in collaboration with the international organizations. The NBC have established the collaboration with the Quantum Pharmaceuticals Pty Ltd in Australia and Nimura Genetic Solutions, a Japanese branch in Malaysia. The MSP collaborated with the University of Wollongong (UOW) and James Cook University (JCU) in Australia, and the BIOTEC in Thailand. The MSP projects, where the author is fully involved, achieved two objectives: a) discovered new drug lead compounds and b) validated the claim of the BTM uses providing scientific basis for those studied plants.

In between 2002 and 2004, four medicinal plants: *Aconitum orochryseum, Corydalis calliantha*, *Tribulus terristris* and *Ranunculus brotherusi* were studied for their phytochemicals and pharmacological activities [5] jointly by the MSP, UOW (Australia) and BIOTECH in Thailand. Three new and two known diterpenoid alkaloids were discovered from *A. orochryseum* [31] and one of these alkaloids, atisinium chloride was identified as the drug lead compound against the multi-drug resistant *Plasmodium falciparum* malaria [32]. Similarly, four known isoquinoline alkaloids with antimalarial activities were isolated from *C. calliantha* where cheilanthifoline showed best *in vitro* activities against the *P. falciparum* chloroquine and antifolate sensitive TM4 strain with IC_50_ values of 0.90 μg/mL and multi-drug resistant K1 strain with IC_50_ values of 1.22 μg/mL [33]. This alkaloid was also identified as the potential antimalarial drug lead.

In between 2010 and 2014, seven medicinal plants: *A. laciniatum, Ajania nubigena, Codonopsis bhutanica, C. crispa, C. dubia, Meconopsis simplicifolia* and *Pleurospermum amabile*; were studied for their drug lead compounds [34]. Employing the ethno-directed bio-rational approach, the biological activities of the crude extracts and the isolated compounds were first determined *in vitro*. Out of seven plants that were studied, the crude extracts of the five medicinal plants showed significant anti-malarial activities against the multi-drug resistant *P. falciparum* strains, one plant showed good cytotoxicity against the mammalian KB cells, two plants exhibited fair anti-*Trypanosoma brucei rhodesiense* activity, six plants exhibited moderate TNF-α inhibitory activities and all of the seven plants exhibited mild antimicrobial activity [1, 35].

Inspired by these results, six of these bioactive plant extracts were further analyzed for their phytochemicals and biological activities which resulted in the identification of 172 phytochemicals, of which 46 compounds were isolated as alkaloids, terpenoids, furanocoumarins and flavonoids using ESI-MS, GC-MS, NMR, IR and the single crystal X-ray structural analysis [34]. Out of 28 compounds that were investigated for their biological activities, 13 exhibited positive results and four of them: protopine, scoulerine, simplicifolianine, and luteolin-7-*O*-*β*-D-glucopyranoside; showed strong anti-malarial activities against multidrug resistant *P. falciparum* strains: TM4/8.2 and K1CB1 which were identified as new drug leads [34]. An antimalarial patent has been obtained for one of the new alkaloid, simplicifolianine which was isolated from *M. simplicifolia* [36]. Protopine was isolated from *C. crispa* [37], luteolin-7-*O*-*β*-D-glucopyranoside from *A. nubigena* [23] and oxypeucedanin methanolate which had moderate antimalarial activity was isolated from *P. amabile* [38, 39]. An alkaloid-A1 isolated from *C. dubia* (under investigation), showed significant inhibition of the acetylcholinesterase with a minimum inhibitory requirement of 0.0015 nmol (A1), which is almost two-fold better than the galanthamine (0.003 nmol)-a drug currently used for treating Alzheimer’s disease [40].

**Fig. 4:**
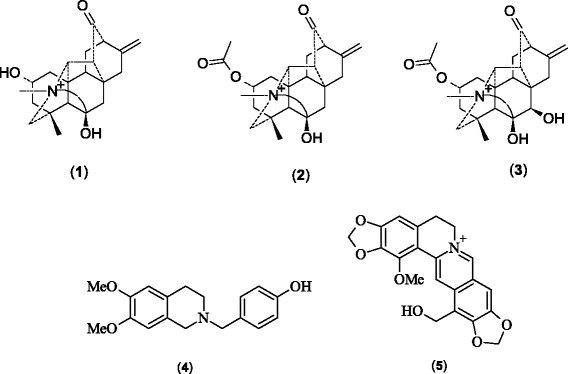
Structure of new phytochemicals isolated from the Bhutanese medicinal plants

These biodiscovery studies also discovered five new phytochemicals (Fig. 4) from three medicinal plant species: *A. orochryseum*, *C. dubia* and *M. simplicifolia*. Orochrine (**1**), 2-*O*-acetylorochrine (**2**) and 2-*O*-acetyl-7α-hydroxyorochrine (**3**) were isolated from *A. orochryseum* [5]. Dubiamine (**4**) was isolated from *C. dubia* [40] and simplicifolianine (**5**) from *M. simplicifolia* [36]. The new compounds were named after their species as: orochrine based on *orochryseum*, dubiamine based on *dubia* and simplicifolianine after *simlicifolia*.

Overall, these studies provided the scientific evidences to the ethnopharmacological uses of the Bhutanese Himalayan medicinal plants and identified the new drug lead compounds thereby demonstrating the effective role of medical ethnobotany in the drug discovery initiatives in Bhutan. The financial return from the biodiscovery projects are not guaranteed and the average duration for the drug development involving clinical trials takes about 8–12 years. However, given that the country still has 2/3 of her biodiversity intact and that it has huge bioprospecting potential, there is no reason why Bhutan cannot pursue properly planned biodiscovery programs that could bring in million dollar revenues if one new drug can be discovered.

### Challenges of the medicinal plants, BTM and the biodiscovery programs

The medicinal plants programs have huge socio-economic bearing including free health care and traditional medical education, income generation, and the preservation of traditional medical heritage and environment. The main challenge for the MSP has been securing constant supply of medicinal plants. Whole activities depend on the medicinal plants and any mismanagement brews collapse of the whole fabrics of the BTM system. The guidelines for identification and collection of medicinal plants in Bhutan [41] states that the current utilization of medicinal plants by the MSP is under sustainable range. However, expansion of the traditional health care units from the current 48 BTM units to cover all the 178 Basic Health Units (as planned by the government) in the country, will require 4 times more medicinal plants supplies to meet the medicine production demand. This would put pressure on the medicinal plants and their environment if proper management plans are not laid down. The rising herbal industries including incense manufacturers and the illegal networks of herbal traders already adds to this problem. The domestication and cultivation trials of some of the endangered species are yet to bear fruit. The dilemma is whether to expand the traditional medical health care services to the people at the expense of long term threat to the sustainability of the medicinal plants and their environment. The balancing act in these regard has been very challenging even at the current scenario.

For BTM, the challenge stems from the manufacturing aspects in terms of maintaining the quality of the medicines. The preparation of the polyherbal formulations encounters the following issues: a) some of the ingredients are becoming rare to find and its exclusion from the prescribed formulations could compromise the quality of the polyherbal formulations, and b) the cost of manufacturing the polyherbal TM is rising and few products have even become more expensive than the modern allopathic drugs thereby undermining the affordability to manufacture them by the MSP. In addition, due to exorbitant price fetched by the *Cordyceps sinensis* in the open market, the interest of some of the highland yak-herders in collecting other medicinal plants (sold solely to MSP by the collectors) has been dwindling over the years. Rewiring their interest would mean hiking the prices of the medicinal plants, which would shoot up the production cost to even higher level. Since some of the polyherbal formulations contain as much as 35 ingredients, developing appropriate quality parameters and assessing their quality including the identification of the marker compounds and the biological activities becomes complex, difficult to achieve and costly. In general, the quality, safety, and effectiveness of the multi-ingredient formulations depend on the quality of their source materials and how they are handled through collection and the production processes. Therefore, much impetus has been given to first address the quality of the source materials or individual plants that make up the more complex polyherbal formulations. Even doing this involves research protocols developed for modern medicines, which often does not suit the complexities of the BTM. Whatever the research that has been carried out at MSP so far has focused on surveying and authentication of medicinal plant species, documenting folklore medicines (ethnomedicines), developing monograph and the product profiles, establishing quality control parameters and daily monitoring of both raw materials and finished products through basic analytical methods, and standardization of the production processes.

The biodiscovery initiatives under the government ownership have begun but has long way to go. Carrying out good and comprehensive scientific biodiscovery research requires adequate laboratory infrastructure, financial resources and the multi-disciplinary approach involving different expertise including the traditional medical practitioners, ethnobotanists, pharmacognosists, phytochemists, pharmacologists and modern doctors. Some of these has been lacking at both the MSP and the NBC. Moreover, being a Buddhist country, there is a strong culture of respect for animals. Conducting animal studies, which is an essential component of biodiscovery, have been not well received by the BTM practitioners in Bhutan. It is challenging to involve the *Drungtshos* (traditional physicians) in research as they are the practitioners of BTM that has so much to do with Buddhism, which requires oath taking for not to kill but to save all forms of lives including human and animal. Thus, collaboration is the best strategy for successful implementation of biodiscovery projects in Bhutan.

## Conclusion and future directions

It is understood that the medicinal plants program is one of the sustainable vehicles of GNH. Given that there is good government support and dynamic governance in this area, other pillars of medicinal plants-based GNH including preservation of rich traditional medical culture, conservation of medicinal plants and their environment, sustainable socio-economic development and biodiscovery would all fall in place. Despite numerous challenges, the medicinal plants are seen as a golden goose for the farmers that helps them in generating income and overturning their poverty. For patients, its formulations or medicines is a solace of treatment and cure. For the BTM practitioners, it is a source of employment. For the chemist and researchers, it is a potential chemotherapeutic pool waiting for biodiscovery. Overall, this medicinal plant programs accelerates the community vitality, dynamics and the well-being of the society, and produces prosperity and the happiness. Failing to maintain the sustainable growth and supply of medicinal plants would mean downfall of these whole fabrics of developments. It would negatively affect the networks of medicinal plants stakeholders including patients, farmers, brokers, business people, buyers, MSP, TMH, FTM, MAPS and the NBC. Therefore, it is vital to maintain, manage and utilize the medicinal plants wisely and have proper long-term sustainable management plan.

While the current consumption of medicinal plants in Bhutan is considered under sustainable range, there does not exist proper research data that suggest their long-term sustainability. Since the yak-herders and famers have already expressed the difficulties to collect some of the medicinal plants in required quantities by MSP, urgent research to analyze this issue and understand the impact of current collection practices in the existing collection centers is quintessential. The MSP, MAPS and the NBC have done sporadic surveys including the value chain analysis of few plant species and LAMP surveys in selected districts. However, there is need for conducting more systematic medicinal plants survey covering whole country which could facilitate the establishment of the new alternative collection sites for MSP wherein the farmers are the benefactors. These suggested studies and survey findings would help the government and the policy makers in drawing proper management plans for the medicinal plants and also in regulating the number of the establishment of herbal industries in the country. Although, higher the numbers of herbal industries mean creating more businesses and jobs, it should not come at the cost of over farming or depleting medicinal plants and their environment.

Biodiscovery research and quality assurance of the medicinal plants has been the focus of discussions both within the MSP and the Ministry of Health. The MSP and the NBC have the basic analytical laboratory (funded by the government) to initiate the preliminary biodiscovery projects. However, division of financial resources between these two organizations have limited the growth of advanced research laboratory and the technically qualified human resource development. Assimilating the efforts, financial resources and the research facilities of those two organizations or combining them and establishing as one government-owned autonomous Biotechnology laboratory would make the biodiscovery initiatives more efficient and cost effective. There is need to expand and enhance research collaborations especially with the regional WHO recognized traditional medical research centers and with other biodiscovery research organizations. Even big universities and the research institutes rely on strong collaborative approach, as the biodiscovery research requires multi-disciplinary expertise and facilities that are very difficult to establish and harmonize in just one organization owing to capital intensiveness.

However, while looking for the collaboration partners, the most difficult process is the intellectual property rights protection. The NBC and the Intellectual Property Rights Division under the Ministry of Trade and Industry have developed the acts and other legal frameworks pertinent to the use of natural resources and biodiversity, patenting and ownership. The recent Biodiversity Action Plan-2009 [5], which is a built up of the previous acts of 1998, 2002 and 2003; places greater emphasis on protecting farmers’ rights and guarantees equitable benefit sharing from the use of the resources including biodiscovery from medicinal plants. Bhutan is also signatory to the United Nations Convention on Biological Diversity. Governed by these acts, the MSP and the NBC have successfully completed few rounds of biodiscovery collaborations with the selected universities and pharmaceutical companies. For example, the 2010–2014 (4 years duration) collaboration coordinated by the author between the MSP and NBC in Bhutan, UOW and JCU in Australia, and the BIOTEC in Thailand have been successfully completed and landed with two patent applications. Collaboration using similar model can be implemented in Bhutan.

In summary, while the BTM and the medicinal plants programs have significantly contributed to the growth of economic, health and wellbeing of the people, it can be enhanced through immediate attention to the following:

- Assessment of the impact of current harvesting practices to the medicinal plants.
- Further surveys of medicinal plants across Bhutan, to more thoroughly understand their distribution, abundance and ecology.
- Develop management plans for all the wild types of medicinal plants in order to help regulate their long term sustainability.
- Enhance medicinal plants cultivation trials to conserve uncommon species. Cultivating all the imported LAMP within Bhutan can generate more income to the farmers and therefore requires urgent attention.
- Expand and enhance research collaborations to facilitate further scientific studies and biodiscoveries.
